# γ-tubulin as a signal-transducing molecule and meshwork with therapeutic potential

**DOI:** 10.1038/s41392-018-0021-x

**Published:** 2018-09-14

**Authors:** Maria Alvarado-Kristensson

**Affiliations:** Molecular Pathology, Department of Translational Medicine, Lund University, Skåne University Hospital, Malmö, 20502 Sweden

## Abstract

Knowledge of γ-tubulin is increasing with regard to the cellular functions of this protein beyond its participation in microtubule nucleation. γ-Tubulin expression is altered in various malignancies, and changes in the *TUBG1* gene have been found in patients suffering from brain malformations. This review recapitulates the known functions of γ-tubulin in cellular homeostasis and discusses the possible influence of the protein on disease development and cancer.

## Cancer: Cytoskeleton regulator identified as promising drug target

The association of γ-tubulin with tumor growth suggests that γ-tubulin inhibitors could be used as anticancer therapeutics. γ-tubulin is a member of the tubulin family that is best known for regulating the assembly of microtubules, part of the cell’s cytoskeleton. Maria Alvarado-Kristensson at Lund University, Sweden, reviews recent studies on γ-tubulin-rich structures that form a meshwork in both the cell cytoplasm and nucleus. These structures not only ensure the proper positioning of organelles but also that key signaling proteins are in the right place at the right time for cell cycle progression. High levels of γ-tubulin are found in various tumors and compounds that target γ-tubulin activity reduce tumor growth in animal models. Further work will determine whether drugs that target γ-tubulin will be more effective and have fewer side effects than those developed to target α- and β-tubulin.

## Introduction

**Fig. 1:**
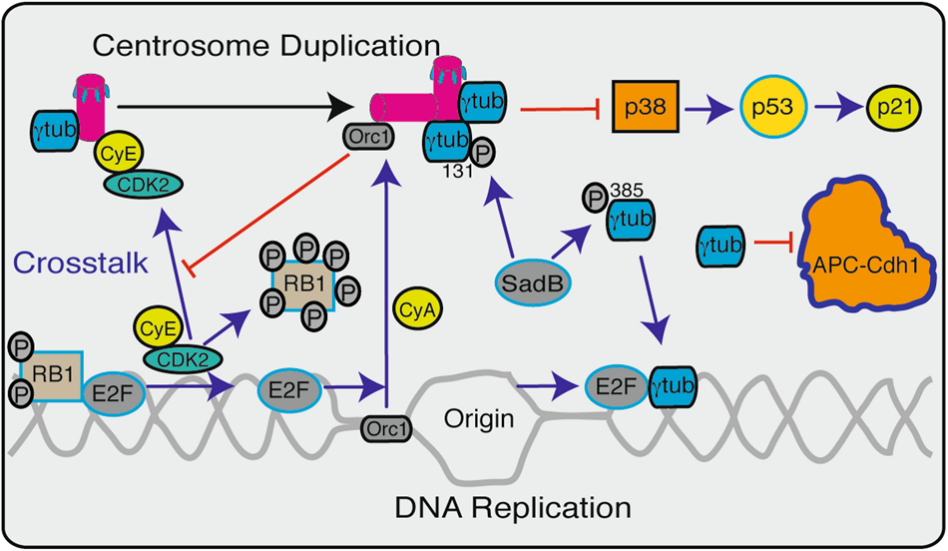
Schematic representation of the different ways in which γ-tubulin (γtub) and the centrosomes control the G1-to-S transition and S phase progression. The blue and black arrows indicate centrosome activation and duplication, respectively, and the red lines indicate inhibition. At the G1–S transition, SadB mediates the phosphorylation of γ-tubulin on Ser^131^ and Ser^385^, and this action regulates the recruitment of γ-tubulin to the growing centrosome and leads to the nuclear accumulation of γ-tubulin. The centrosomes inhibit the activation of the p38 mitogen-activated protein kinase (p38)-p53-p21 signal pathway. The centrosomal localization of cyclin E (CyE)–cyclin-dependent kinase (Cdk2) is required for the initiation of DNA replication. Once DNA replication is initiated, the origin replication complex subunit 1 (Orc1) translocates from the origin of replication to the centriole in a cyclin A (CyA)-dependent manner, where it prevents the CyE-dependent reduplication of the centrosomes. To progress through the S phase, the CyE–Cdk2 complex phosphorylates the protein retinoblastoma 1 (RB1), and this initiates the transcriptional activities of E2 promoter binding factors (E2Fs). The activities of the E2Fs trigger the initiation of centrosome duplication and DNA replication, and the accumulation of γ-tubulin in the nuclear compartment turns off the transcriptional activities of E2Fs. Additionally, γ-tubulin inactivates the anaphase-promoting complex/cyclosome^Cdh1^ at the G1-to-S transition

**Fig. 2:**
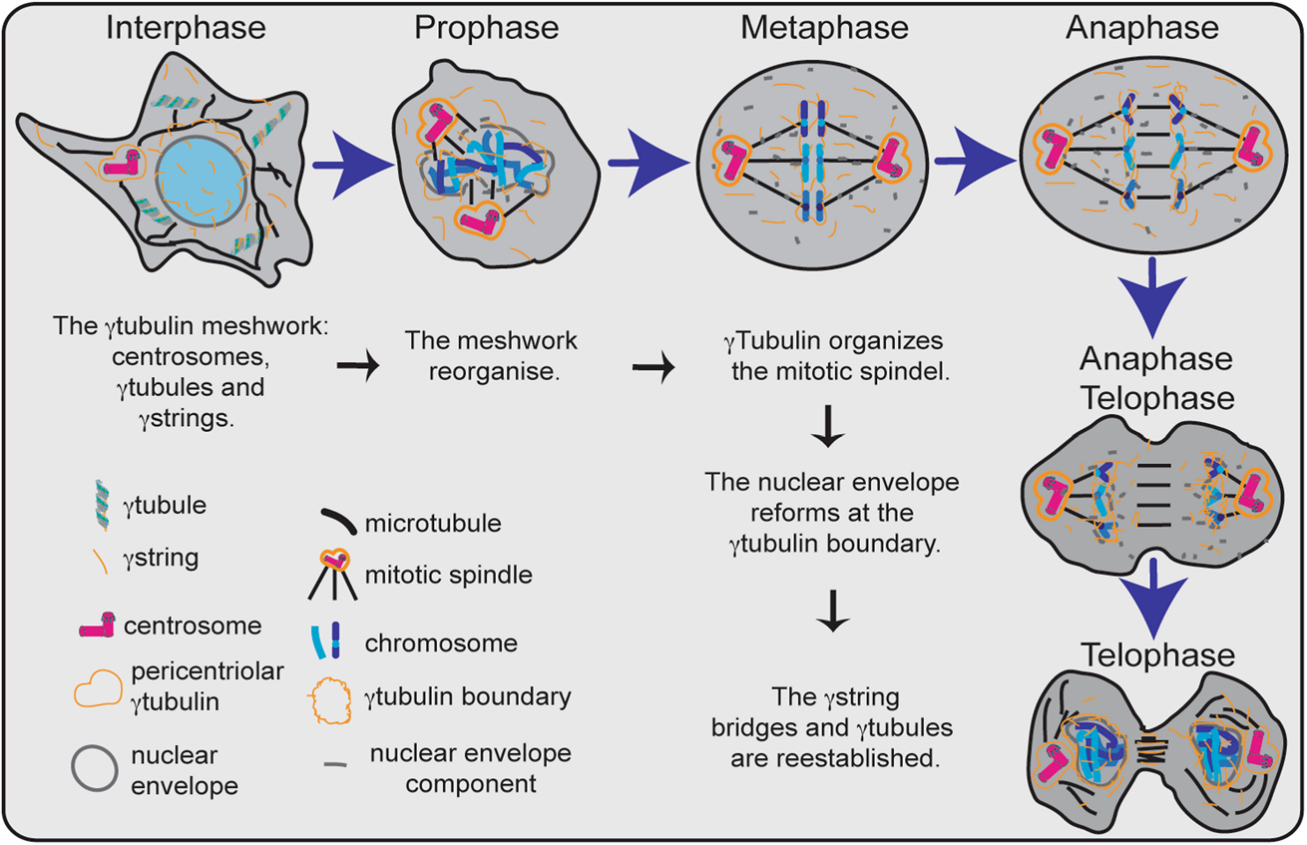
Hypothetical representation of the functions performed by the γ-tubulin meshwork during interphase and mitosis. In interphase, the meshwork is composed of centrosomes, γ-tubules, and γ-strings. The nuclear and the cytosolic pool of γ-tubulin are connected with γ-strings across the nuclear envelope. γ-tubules regulate the concentration of the cytosolic pool of γ-tubulin. The γ-tubulin meshwork changes in a cell-cycle-dependent manner. In prophase, the nuclear envelope is dispersed. γ-tubulin accumulates in the pericentriolar region of the centrosomes and assists in formation of the mitotic spindle. The number of γ-tubules is reduced in mitotic cells (prophase, metaphase, anaphase, and telophase). During mitosis, the centrosomes coordinate the segregation of chromatids between the newborn cells through orientation and organization of the mitotic spindle. The dispersed components of the nuclear envelope localize to the mitotic spindle and cell periphery. In anaphase/telophase, the components of the nuclear envelope nucleate at the γ-tubulin boundary. At the end of mitosis, the nuclear envelope is formed, and the γ-string bridges are reestablished

The essence of life is the self-renewal ability of cells. The birth of a cell is the result of a dynamic process in which proteins execute a reproduction program that is encoded in the DNA. In eukaryotes, this program is supported by energy obtained through compartmentalization within individual cells, which creates the need for transport and communication (signal transduction) between the intracellular compartments.^1^ The dynamics in this context are achieved by cytoskeleton and nucleoskeleton networks that provide the form and mechanical support for a cell, organize the genome, and assist in signal transduction, cell movement, cellular transport, and cell–cell interactions.^2^ The various cytoskeletal networks (i.e., actin, microtubules, and intermediate filaments) can perform transport through cellular sensory sites, such as focal adhesion macromolecules and linkers of nucleoskeleton and cytoskeleton (LINC) complexes, and in this manner transmit and coordinate a cellular response to environmental queue signals.^3^ These sensory sites are important signaling hubs that integrate skeletal and signal transduction proteins. Other sensory hubs include the centriole/basal body and centrosomes. A centriole is composed of a cylindrical array of microtubules.^4^ In dividing cells, two centrioles are embedded in a pericentriolar protein matrix (PCM) to form a centrosome. The PCM constitutes an important site that regulates microtubule nucleation and dynamics. During cell division, the link between the two centrioles is lost in the M to G1 phase transition, which allows for the replication of the centrioles. In the S phase, the inherited centrosome and genome duplicate synchronously (Fig. 1). At the onset of mitosis, the two centrosomes ensure the assembly of a bipolar mitotic spindle and the strict segregation of sister chromatids between daughter cells (Fig. 2). At the end of cell division, a mammalian daughter cell inherits one centrosome and one genome set. In yeast, the formation of microtubule-organizing centers at the telomeres induces meiotic telomere clustering.^5^ Together, these observations suggest that centrosomes are involved in coordinating chromosomal movements.

In resting cells, flagella, or cilia can extend from the centrioles, and such centrioles are therefore usually referred to as basal bodies.^4^ In both cases, a centrosome and a basal body link intermediate filaments, microtubules, and components of the actin network with signal transduction molecules.^6–9^ The functions of cilia and flagella are cell-type specific, but in general these organelles enable a cell to move itself or to transport fluids across the cell surface, sense environmental cues, and transmit signals to the cell interior. By doing so, cilia and flagella control our bodily processes during both development and tissue and organ homeostasis.^10,11^ Defects in the sensory and/or motor functions of cilia and flagella are involved in severe human diseases and syndromes, such as polycystic kidney disease (PKD), primary cilia dyskinesia, retinal degenerative disease, planar cell polarity, and Kartagener’s syndrome (*situs inversus*, sinusitis, and bronchiectasis), as well as developmental diseases grouped within the Bardet-Biedl syndrome, which include obesity and diabetes.^11^

## Tubulins

**Fig. 3:**
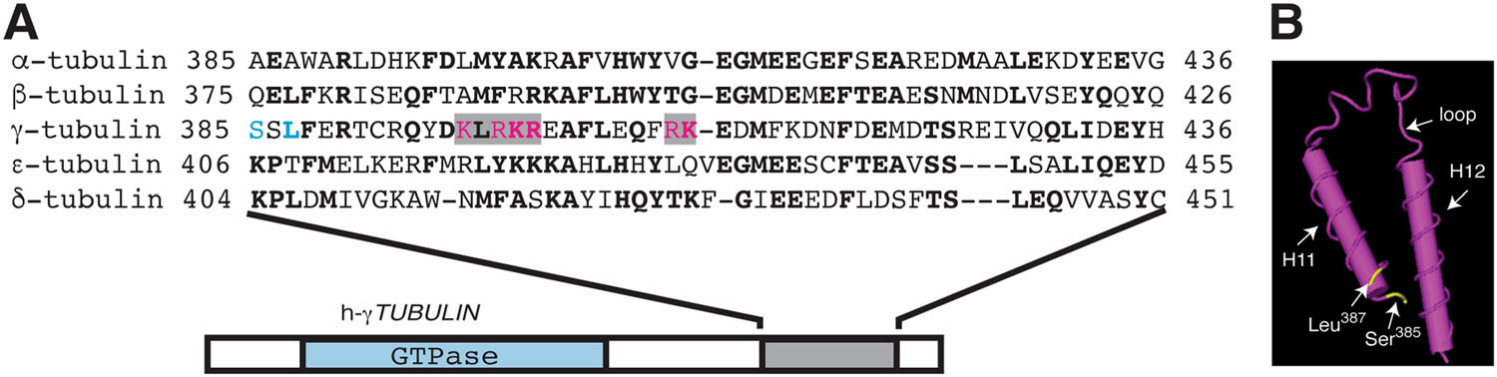
The C terminus of γ-tubulin contains the DNA-binding helix-loop-helix motif. **a** Sequence alignment of the C-terminal helix (H11)-loop-helix (H12) region of human γ-tubulin 1 (residues 385–436; corresponding to helix numbers 11 and 12 in the γ-tubulin protein)^95^ and α- (residues 385–436), β- (residues 375–426), ε- (residues 406–455), and δ-tubulin (residues 404–451). Bold letters indicate identical residues. The bipartite nuclear localization signal (NLS) of γ-tubulin is highlighted in gray, and the magenta letters represent residues included in the NLS. Ser^385^ and Leu^387^ in γ-tubulin are labeled in blue. **b** The known three-dimensional structure of the C-terminal helix-loop-helix region of human γ-tubulin revealed with the three-dimensional structure viewer Cn3D.^95^ In the structure, the SadB putative phosphorylation sites at Ser^385^ and Leu^387^ are depicted in yellow. The phosphorylation of Ser^385^ leads to accumulation of γ-tubulin in the nuclear compartment. Mutations in Leu^387^ have been found in patients suffering from lissencephaly and microcephaly

Microtubules and centrosomes are highly enriched in a family of GTPases called the tubulins.^12^ In humans, there are five known tubulin isoforms: α-tubulin, β-tubulin, γ-tubulin, δ-tubulin, and ε-tubulin.^12^ In the tubulin family, the C-terminal region is the most variable area (Fig. 3a); whereas, the GTPase domain in the N-terminal region is more conserved. The protein sequence in the N-terminal region (γ-tubulin residues 1–333) of γ-tubulin exhibits 56 and 60% homology with the corresponding sequences in α- and β-tubulin, respectively.^13,14^ In contrast, the homologies of the C-terminal region (γ-tubulin residues 334–451) of γ-tubulin are 48% with α-tubulin and 50% with β-tubulin.

In eukaryotic cells, a microtubule usually consists of 13 laterally associated protofilaments that form hollow tubes in the cytoplasm, axons, and mitotic spindles.^15^ Protofilaments are made up of α- and β-tubulin heterodimers, and flagella, cilia, and centrosomes contain various modified protofilament combinations. A microtubule in a centriole displays a ninefold radial symmetry that, in some cases, continues into the flagellum or cilium that emanates from it. The centriole architecture of microtubules is regulated by δ- and ε-tubulin.^4^

The γ-TUBULIN gene was first discovered in the fungus *Aspergillus nidulans* as a regulator of microtubule assembly,^16^ and it is highly conserved among species. Two γ-TUBULIN genes and one pseudogene have been described in humans.^17,18^ Initially, γ-tubulin was considered to be a minimally abundant protein,^19^ but this was a clear underestimation. γ-tubulin is a ubiquitously expressed protein that appears in abundance in both the cytosol and the nuclear compartments of cells in all mammalian tissues.^13,14,20–23^

Together, γ-tubulin and various γ-tubulin complex proteins (GCPs) form a ring-shaped structure in the cytosol called the γ-tubulin ring complex (γTURC) that regulates the nucleation of α- and β-tubulin dimers into unidirectionally growing microtubules.^15^ Additionally, γ-tubulin is enriched at the PCM and plays a role in centriole duplication.^24–26^

## The cellular organization of γ-tubulin

Three main experimental set-ups have been applied to investigate the cellular function of γ-tubulins. The first of these methods is the widely used siRNA-mediated reduction of the γ-tubulin pool, which partially decreases the amount of cellular γ-tubulin. The second approach is the introduction of mutations into the protein that only affect a targeted function.^13,14,23,27–29^ The third strategy is the use of extracts from *Xenopus* eggs;^30^ in short, the egg extracts are combined with exogenously added DNA, and a reduction of γ-tubulin in the extracts is achieved by immunodepletion, although depletion of the DNA has not been performed in most experiments.^30,31^ Nonetheless, despite a partially reduced γ-tubulin pool, most studies have been performed in the presence of the protein, which has led to weak or null phenotypes. Consequently, all of the functions of γ-tubulin have not yet been identified.

At the cellular level, simultaneous knockdown of both γ-TUBULIN genes is lethal.^32–34^ γ-TUBULIN 1 knockout mice survive only to the morula/blastocyst stage because redundant function is provided by the activation of γ-TUBULIN 2 during the first stage of embryonic development.^33^ In contrast, γ-TUBULIN 2 knockout mice are viable and fertile.^33^ In human neuroblastoma cell lines, both neuronal development and mitochondria-induced oxidative stress result in the upregulation of γ-TUBULIN 2, which is considered to represent a pro-survival signal.^35^

Due to its abundance, cellular γ-tubulin is organized in a variety of structures. Part of the cellular pool is folded into γ-tubulin threads called γ-strings by the type II chaperonin CCT.^36^ γ-Strings are 4–6 nm in diameter^23,28,36,37^ and span from the cytosolic compartment through the nuclear membrane and into the chromatin. A γ-string is very thin and alone can offer little stability. However, when γ-strings occur together in large numbers in association with cellular membranes, they may reinforce and stabilize those membranes (Fig. 2). In support of this conclusion, the proper positioning and biogenesis of the Golgi apparatus depend on the interaction of γTURC with the Golgi membrane-linked GMAP-210 protein.^38^ Furthermore, it was recently reported that γ-tubulin becomes associated with endosomes^39^ and that γ-tubulin is an important mitochondrial structural component that maintains the mitochondrial network to provide the mitochondria with a cellular infrastructure.^23,35^ Similarly, the numerous DNA-bound γ-strings anchor the nucleus to the cytosol, and the transition between cytosolic and nuclear-associated γ-strings aids the formation of a nuclear envelope around the chromatin (Fig. 2).^28,40–42^

In addition to γ-strings, γ-tubulin forms cytosolic fibers called γ-tubules that are 20–25 nm in diameter (Fig. 2).^34^ In contrast to γ-strings, which are static structures, γ-tubules are temperature-sensitive polar structures that vary both in size and location and can emanate from centrosomes.^34^ γ-tubules differ from microtubules in that they consist of pericentrin and γTURCs, and they lack α- and β-tubulin heterodimers.^34^ Other γ-tubulin-rich structures include the centrosomes. This picture implies that the γ-tubulin and associated proteins in the centrosomes, the γ-tubulin in the γ-strings, and the γ-tubulin and associated proteins in the γ-tubules link together to form a cellular meshwork in both the cytosol and the nuclear compartment (Fig. 2).

## The γ-tubulin meshwork as a signal transducing platform

There is substantial evidence that the centrosomes and γ-tubulin regulate the G1-to-S transition,^13,14,43–51^ mitotic progression,^52^ and cytokinesis.^53^ These effects are achieved partly through timely changes in the location of γ-tubulin that occur in a manner that is related to cell division. In the G1 phase, most of the γ-tubulin pool is in the cytosol, and part of this cytosolic pool creates γ-tubules (Fig. 2).^34^ However, at the G1–S transition, the number of cytosolic γ-tubules is reduced, and there is a subsequent accumulation of γ-tubulin in the nuclear compartment and the PCM.^14,24,34^ During cell division, the PCM acts as signal hub that brings together various checkpoint proteins. Indeed, the PCM content changes in a cell-cycle-dependent manner and harbors various proteins involved in the signal transduction pathways that coordinate cell cycle progression and function as checkpoints. Removal or disruption of the centrosomes impairs cytokinesis and thereby causes G1 arrest,^44,53,54^ which might be induced by the p38-p53-p21 signal pathway (Fig. 1).^48^

Some of the signals that synchronize centrosome duplication with DNA replication arise from signal transduction events that occur at the centrosomes (Fig. 1). The centrosomal localization of cyclin E-Cdk2 is required for the initiation of DNA synthesis in CHO-K1 cells.^50^ Once DNA replication and centrosome duplication are initiated, the origin replication complex subunit Orc1 translocates from the origin of replication to the growing centrosome in a cyclin-A-dependent manner, and, in that location, Orc1 prevents cyclin-E-dependent reduplication of the centrosomes (Fig. 1).^55^ Moreover, early in the S-phase, SadB kinases (e.g., mSADB and hSAD1/BRSK1) mediate the phosphorylation of γ-tubulin on Ser^131^ and Ser^385^.^24,27,56^ The phosphorylation levels of γ-tubulin on Ser^131^ regulate the recruitment of γ-tubulin at the nascent centriole and facilitate the accessibility of SadB to Ser^385^. The latter phosphorylation site is near the nuclear localization signal (NLS) of γ-tubulin and induces a conformational change to unmask the NLS, which leads to the nuclear accumulation of γ-tubulin (Figs. 1 and 2).^13,14,20–22,24,27^ This process implies that the cytosolic and nuclear γ-tubulin pools have different conformations, which could provide the basis for the development of therapeutic compounds that target the nuclear activity of γ-tubulin. Furthermore, studies of murine NIH3T3 embryonic fibroblasts and human U2OS osteosarcoma cells have indicated that γ-tubulin is the only member of the tubulin family that has a bipartite NLS on the C terminus (Fig. 3).^27^ Notably, Ser^385^ is located at the starting region of a motif that is commonly found in DNA-binding proteins called a helix-loop-helix (Fig. 3b). This motif concurs with the DNA-binding ability of the C terminus of γ-tubulin.^14^

To enable the G1-to-S phase transition and cell cycle progression, in late G1, the protein retinoblastoma 1 (RB1) is inactivated to initiate the transcriptional activities of E2 promoter binding factors (E2Fs). The transcriptional activities of E2Fs are necessary to induce the expressions of target genes that are essential for centrosome duplication and DNA replication.^57^ In the S phase, nuclear γ-tubulin turns off the transcriptional activities of E2Fs (Fig. 1).^13,14,43^ Furthermore, γ-tubulin inactivates the anaphase-promoting complex/cyclosome^Cdh1^ at the G1-to-S transition (Fig. 1).^51,58^

It is not only the presence or absence of centrosomes that affects cell division. In *Drosophila*, this process is regulated by the positioning of the centrosomes in germline stem cells,^49^ and the Rab11-mediated association of γ-tubulin with endosomes contributes to the organization and orientation of mitotic spindles.^39^ Moreover, the centrosomes contain various protein kinases that are involved in mitotic progression.^59,60^ During mitosis, γ-strings assist in the formation of the nuclear envelope around chromatin,^28,40–42^ whereas γTURCs regulate microtubule nucleation and mitotic progression.^29,52,61–63^

In addition to interacting with proteins that are involved in microtubule nucleation and cell cycle progression, γ-tubulin associates with Rad51, C53, BRCA1, p53, Chk2, and ATR,^21,22,59,64–69^ which are proteins that are involved in checkpoint activation and DNA repair. However, the involvement of γ-tubulin in DNA repair remains to be elucidated. It is plausible that the γ-tubulin meshwork could serve as a signal transduction hub that coordinates various cellular responses.

## Role of γ-tubulin in disease

Thus, γ-tubulin is associated with various checkpoint proteins and plays a role in regulating cell division, which suggests that the γ-tubulin meshwork is involved in cancer development.^21,22,59,64–69^ Moreover, centrosome amplification is correlated with high-tumor histological grade, lymph node metastasis, and poor prognosis.^70–72^ Furthermore, in various tumors and cell lines (medulloblastoma, myelomas, non-small cell carcinoma, breast cancer, gliomas, and glioblastoma), the localization pattern and expression levels of γ-tubulin are altered.^73–78^ Those observations imply that increased levels of γ-tubulin may lead to a more complex γ-tubulin meshwork and thereby favor tumor progression.

Intriguingly, in various tumors (i.e., retinoblastoma and bladder, breast, colorectal, and small cell lung carcinomas [SCLCs]), γ-tubulin and RB1 moderate each other’s expression, and, in the absence of γ-tubulin and RB1, the uncontrolled transcriptional activities of E2Fs upregulate apoptotic genes that cause cell death.^13,14^ In some tumor types, the *RB1* gene is deleted or carries somatic mutations. Furthermore, the RB1 pathway is frequently silenced in multiple malignancies, such as prostate, bladder, colon, small cell lung, and breast cancers and in retinoblastoma osteosarcoma, neuroblastoma, and lymphoblastic leukemia.^13,43,79–84^ Thus, the inhibition of γ-tubulin has been suggested as a strategy for broad-range targeted anti-cancer therapy for RB1-deficient tumors.^13,14,43^ Indeed, the citral analogue citral dimethyl acetal (CDA) and the approved drug dimethylfumarate (DMF) target the nuclear activity of γ-tubulin, and both of these agents exhibit antitumorigenic activity in vivo.^43,85^ Notably, DMF has been approved by the FDA for the treatment of multiple sclerosis and psoriasis and has few side effects when used for these purposes.^86,87^ In contrast, the microtubule-depolymerizing drugs colchicine and gatastatin affect the functions of γ-tubulin,^34,88,89^ but these agents have numerous side effects that are related to their depolymerizing influence on the microtubules. Colchicine is used to treat gout and familial Mediterranean fever.^90^

Interestingly, spontaneous mutations in *TUBG1* are associated with lissencephaly and microcephaly, which are two of the most common brain malformations that can lead to mental retardation and neurological morbidity in children.^91,92^ *TUBG1* gene mutations in this context cause changes in the amino acids Tyr92Cys, Thr331Pro, and Leu387Pro. Notably, in yeast cells, the Tyr92Cys mutation affects microtubule positioning; whereas, the Leu387Pro mutation in the DNA-binding domain of γ-tubulin (Fig. 3) influences nuclear positioning, which highlights the importance of the γ-tubulin meshwork in cellular homeostasis and the influence of this network on disease development.

## Conclusion

Our knowledge is limited regarding to the functions of γ-tubulin beyond the role of this protein in microtubule nucleation and possible use as a therapeutic target. This review summarizes the known functions of γ-tubulin in cellular homeostasis, and considers the possibility that this protein has an influence on disease development and can be used in cancer treatment. However, further research is needed to elucidate many aspects of the functions of γ-tubulin and how they are associated with the occurrence of disease, for example, in terms of the immune system and the development and functions of cilia, flagella, and the brain. Today, various chemotherapies are focused on the impairment of microtubule function to reduce tumor growth,^93,94^ and these agents are prescribed for a broad range of malignancies, including lung, breast, gastric, esophageal, bladder, and prostate cancers, Kaposi sarcoma, and squamous cell carcinomas of the head and neck.^94^ Unfortunately, the effectiveness of microtubule-targeting drugs for cancer therapy is limited due to drug resistance and severe side effects in treated patients.^94^ Most microtubule-targeting compounds are inhibitors of α- and β-tubulin.^94^

Accordingly, knowledge of the functions and regulation of the γ-tubulin meshwork in cell division, the immune system, and the development of the brain will pave the way for establishing novel broad-range targeted therapies that cause fewer side effects. Thus, understanding the molecular mechanisms that regulate the dynamics of the γ-tubulin meshwork is clearly a prerequisite for the development of such new drugs.
